# Supplementary material for: Empirical methods for controlling false positives and estimating confidence in ChIP-Seq peaks
Source: BMC Bioinformatics. 2008 Dec 5;9:523. doi: 10.1186/1471-2105-9-523 (PMC2628906; doi:10.1186/1471-2105-9-523)
Supplement: Additional File 1 — A variety of html documents from the USeq web site detailing the available applications, their best usage, output file type descriptions, command line menus, etc. [file 1471-2105-9-523-S1.zip › USeqUserGuides/JavaDoc.zip!/JavaDoc/expr/class-use/ExpressedGene.html]

Uses of Class expr.ExpressedGene


|  |  |  |
| --- | --- | --- |
| **Overview** \| **Package** \| **Class** \| **Use** \| **Tree** \| **Deprecated** \| **Index** \| **Help** | |  |
| PREV   NEXT | **FRAMES**    **NO FRAMES**     **All Classes** |  |


---


## **Uses of Class expr.ExpressedGene**

| Packages that use ExpressedGene | |
| --- | --- |
| **expr** | Classes and tools related to expression array analysis. |

Uses of ExpressedGene in expr

| Methods in expr that return ExpressedGene | |
| --- | --- |
| `ExpressedGene[]` | `CorrelationMaps.makeRankedFloatArrays(ExpressedGene[] ex)`             Makes and places RankedFloatArrays in each ExpressedGene, also nulls the ExpressedGene.values. |
| `static ExpressedGene[]` | `CorrelationMaps.parseGeneFile(java.io.File geneFile)`             Converts a tab delimited file of (name chr start stop val1 val2 ...) into an ExpressedGene[] |
| `static ExpressedGene[][]` | `ExpressedGene.splitByChromosome(ExpressedGene[] exprGenes)`             Splits a ExpressedGene[] by chromosome to ExpressedGene[chrom][lines]. |
| `ExpressedGene[][]` | `CorrelationMaps.window(ExpressedGene[] ex)`             Splits a chromosome of ExpressedGenes into windows based on the windowSize and minNumberGenes |

| Methods in expr with parameters of type ExpressedGene | |
| --- | --- |
| `float` | `CorrelationMaps.averageScores(ExpressedGene[] ex)`             Average scores \* square root of number of genes. |
| `ExpressedGene[]` | `CorrelationMaps.makeRankedFloatArrays(ExpressedGene[] ex)`             Makes and places RankedFloatArrays in each ExpressedGene, also nulls the ExpressedGene.values. |
| `float` | `CorrelationMaps.pairwiseCorrelationScore(ExpressedGene[] ex)`             Average coorelation coefficient \* square root of number of genes. |
| `static ExpressedGene[][]` | `ExpressedGene.splitByChromosome(ExpressedGene[] exprGenes)`             Splits a ExpressedGene[] by chromosome to ExpressedGene[chrom][lines]. |
| `ExpressedGene[][]` | `CorrelationMaps.window(ExpressedGene[] ex)`             Splits a chromosome of ExpressedGenes into windows based on the windowSize and minNumberGenes |

---


|  |  |  |
| --- | --- | --- |
| **Overview** \| **Package** \| **Class** \| **Use** \| **Tree** \| **Deprecated** \| **Index** \| **Help** | |  |
| PREV   NEXT | **FRAMES**    **NO FRAMES**     **All Classes** |  |


---
